# Supplementary material for: Tead1a Initiates Transcriptional Priming Through the TEAD1a/YAP‐Notch1‐Spi1/Cebpα Axis to Promote Neutrophil Fate
Source: Adv Sci (Weinh). 2025 Aug 28;12(41):e05441. doi: 10.1002/advs.202505441 (PMC12591127; doi:10.1002/advs.202505441)
Supplement: Supplementary file 4 — Supplementary Dataset [file ADVS-12-e05441-s004.zip › 3-6.zip!/3-6/02-6.cluster/cluster17/Enrich/ego_BP.pdf]

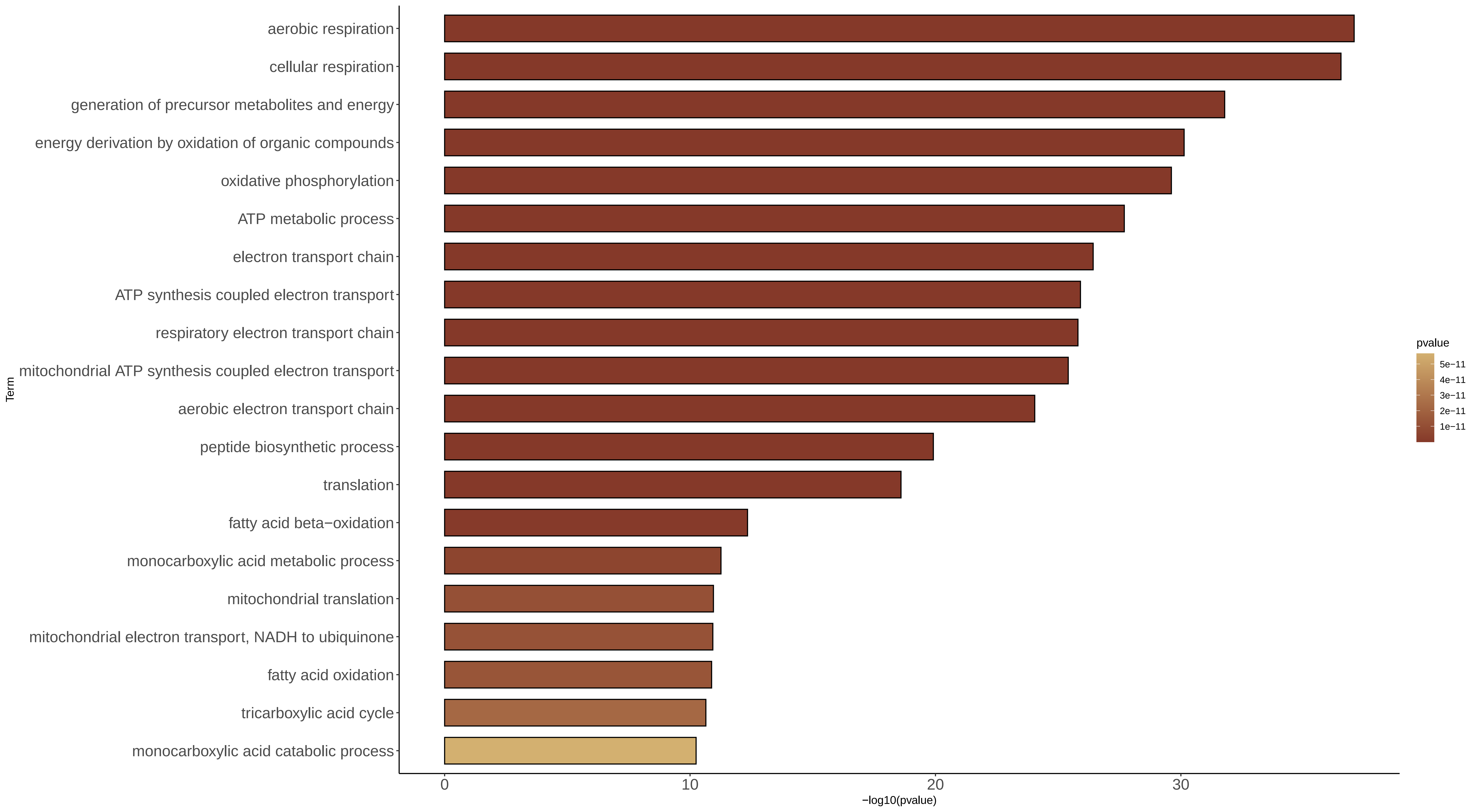

aerobic respiration
cellular respiration
generation of precursor metabolites and energy
energy derivation by oxidation of organic compounds
oxidative phosphorylation
ATP metabolic process
electron transport chain
ATP synthesis coupled electron transport
respiratory electron transport chain
mitochondrial ATP synthesis coupled electron transport
aerobic electron transport chain
peptide biosynthetic process
translation
fatty acid beta−oxidation
monocarboxylic acid metabolic process
mitochondrial translation
mitochondrial electron transport, NADH to ubiquinone
fatty acid oxidation
tricarboxylic acid cycle
monocarboxylic acid catabolic process
Term
0
10
20
30
−log10(pvalue)
pvalue
5e−11
4e−11
3e−11
2e−11
1e−11
